# Kinetics of foot-and-mouth disease vaccine-induced antibody responses in buffaloes (*Bubalus bubalis*): avidity ELISA as an alternative to the virus neutralization test

**DOI:** 10.3389/fvets.2023.1162477

**Published:** 2023-11-07

**Authors:** Juan Manuel Sala, Florencia Celeste Mansilla, María Cruz Miraglia, Sergio Gastón Caspe, Daniel Mariano Perez-Filgueira, Alejandra Victoria Capozzo

**Affiliations:** ^1^Estación Experimental Agropecuaria, Instituto Nacional de Tecnología Agropecuaria (INTA), Mercedes, Corrientes, Argentina; ^2^Instituto de Virología e Innovaciones Tecnológicas, Centro de Investigaciones en Ciencias Veterinarias y Agronómicas (CICVyA), INTA-Consejo Nacional de Investigaciones Técnicas (CONICET), Hurlingham, Buenos Aires, Argentina

**Keywords:** antibody avidity, antibody response, buffaloes, foot and mouth disease virus, maternal immunity, post-vaccination monitoring, serology

## Abstract

The role of water buffaloes in foot-and-mouth disease (FMD) epidemiology as one of the major hosts of the virus that can develop persistent asymptomatic infection highlights the importance of sustaining surveillance on the antibody response elicited by vaccination in these animals. There is gap in the knowledge on how serological assays that measure antibodies against capsid proteins perform with buffalo samples and which would be the most reliable test to substitute the virus neutralization test (VNT) a cumbersome and low-throughput tool for field surveillance. Alternatively, the liquid-phase blocking sandwich ELISA (LPBE) is commonly used. Previous data from our laboratory demonstrated that the vaccine-induced antibodies assessed by the LPBE yielded low specificity with buffaloes’ samples. In contrast, a single-dilution avidity ELISA (AE) aimed to detect high-avidity antibodies against exposed epitopes, combined with an indirect ELISA (IE) to assess IgG levels, produced more reliable results. Here we analyzed for the first time the kinetics of the antibodies induced by vaccination in two different buffalo herds (*n* = 91) over 120 days using AE, IE, LPBE, and the VNT. Kinetics were similar in the different assays, with an increase of antibodies between 0- and 14-days post-vaccination (dpv) which were maintained thereafter. VNT and AE results were concordant (Kappa value = 0.76), and both assays revealed a decay in the antibody response in calves with maternal antibodies at 90 and 120 dpv, which was not evidenced by the LPBE. These results show that kinetics of antibody responses to FMD vaccination are similar in buffalo and cattle, and support the use of indirect ELISA assays, in particular Avidity ELISA, as alternatives to the VNT for vaccine-immunity monitoring irrespectively of the animal’s passive or active immune status.

## 1. Introduction

Foot-and-mouth disease (FMD) is a highly infectious viral disease of cloven-hoofed animals with a unique potential for rapid spread and acute development (1), capable of inflicting severe and far-reaching economic consequences to the livestock industry and international trade (2). FMD is caused by the FMD virus (FMDV), a non-enveloped single stranded positive sense RNA virus (3) which belongs to the family *Picornaviridae*, genus *Aphthovirus* (4). Immunization of susceptible species with good quality conventional inactivated vaccines containing the most recent circulating strains is an effective tool to prevent development of the disease (5) and transmission among susceptible animals (6).

Buffalos represent a major natural reservoir of FMDV. African buffaloes (*Syncerus caffer*) can be readily infected by one or more viral strains and naturally infected animals may maintain persistent infections for months and years with mild or even subclinical manifestations (7). A recent report proposed that new virus variants may be produced in buffalo during the prolonged carriage after acute infection, which in turn may spread the disease to susceptible livestock populations (8). Similarly, Asian buffaloes (*Bubalus bubalis*), also known as water buffalos, may develop subclinical and persistent FMDV infections in naïve (8) and vaccinated herds (9). In those reports, the infectious virus was isolated from a higher proportion of water buffalo samples and for a longer duration compared to cattle (9). Serology-positive buffaloes shed live virus that can be recovered in cultured cells (10). Moreover, experimental transmission from persistently infected to naïve animals has been confirmed for African buffaloes (11) highlighting their potential epidemiological relevance (12). Irrespective of this, the supposed risks are extended to other species and may affect foreign trade of animals and derived products from endemic regions. In line with this perception, most endemic countries where buffaloes are important part of their economy, perform compulsory vaccination campaigns, usually surveyed using the same post-vaccination serological assays designed and validated for cattle.

In Argentina, water buffalo (*Bubalus bubalis*), counting approximately 300,000 heads, is the third largest population in America, after Brazil and Venezuela. More than 80% of the buffalo population is found in the North-Eastern provinces of Formosa and Corrientes (13), within the FMD-free with vaccination zone and close to the border with Paraguay, where the latest regional FMDV-type O outbreak occurred in 2011 (14). They are compulsory vaccinated following the same campaign designed for cattle, twice a year during the first 2 years of age, and once a year thereafter (15–17). The vaccine used is oil-based and contains four viral stains (A24/Cruzeiro, O1/Campos, C3/Indaial and A7Argentina/2001). Buffalo herds are sometimes free-range, crossing borders and sharing grazing with other livestock. Due to their location, behavior and particular pathogenesis regarding FMD, proper monitoring of the vaccine-induced immunity in these populations is paramount.

Measuring FMDV-neutralizing antibodies using the virus neutralization test (VNT) is an accurate procedure for assessing vaccine-induced protective antibodies (15). However, the difficulties inherent to the VNT, such as the need for cultured cells, live FMDV and dedicated facilities prevent this low-throughput assay from being suitable for the field assessment of vaccine-induced antibodies, being ELISAs preferred for this purpose. We have demonstrated before that the currently used liquid-phase blocking ELISA (LPBE) yielded very low specificity with buffalos’ serum samples, probably due to the presence of high levels of “sticky” natural antibodies that over-estimate antigen-specific antibody levels, among other issues related to the assay itself (18). Alternatively, aiming to improve the specificity of the antibody assessment, we developed an indirect ELISA (IE) using purified 140S viral particles, which proved to detect IgG mainly directed against capsid-exposed epitopes (15); and an avidity single-dilution ELISA (AE) based on the same indirect design (15), which includes an additional urea washing step to detach weak binders (18, 19). The improved performance of avidity ELISA compared to LPBE was demonstrated by our group (18) and further verified in a recent study using field serum samples from 40 multi-vaccinated buffalos (20).

Post-vaccination serological monitoring following a compulsory vaccination campaign is a critical policy for FMD control in areas free of disease with vaccination. This is particularly relevant for South America, given the need of appropriate risk assessments before stopping vaccination, a current regional aim. In Argentina, curves that correlate total antibody levels with expected percentage of protection (EPP) to the FMD generalization have been built for the different vaccine strains in cattle (sampled at 60 dpv, challenged at 90 dpv using the “Protection against Podal Generalization,” or PPG, method) (20). These curves provide a solid tool for assessing vaccine potency and herd immunity in cattle, considering a cut-off value corresponding to an EPP > 75% (EPP_75_). However, previous studies in our laboratory indicated that LPBE might overestimate FMDV-specific antibody levels in buffaloes. Here we studied the kinetics of anti-FMDV humoral response following vaccination in buffalos, with or without maternal antibodies, and evaluated the concordance between VNT, LPBE titers, total IgG levels measured by the IE, and avidity of antibodies measured by the AE. The aim of this study was to provide evidence of the suitability of indirect and avidity ELISAs as alternatives to the VNT and LPBE for vaccine serological surveillance using buffalo serum samples.

## 2. Materials and methods

### 2.1. Vaccines

Vaccines used for this study were officially approved commercial tetravalent single oil emulsion formulations containing inactivated FMDV strains A24/Cruzeiro, A/Arg/2001, O1/Campos, and C3/Indaial, produced by a local company. The official vaccine approval certifies the lack of FMDV non-structural proteins (NSP), and that the vaccine can induce antibody titers related to an EPP above 75% (EPP_75_) according to local regulations (21). All animals were injected once with a 2 mL vaccine dose, applied subcutaneously in the neck according to the manufacturer’s recommendations and official regulations from the National Agrifood Health and Quality Service (SENASA), Argentina (22).

### 2.2. Animal welfare

Vaccination was performed by personnel of the National Animal Health Authority (SENASA) in the frame of the mandatory FMD vaccination campaign applied in Argentina following current regulations (22). Serum samples (maximum volume 20 mL/animal) were extracted by puncture of the jugular vein using sterile 50 mL syringes with 18 gauge 1.5 to 2-inch needles, transferred to sterile tubes and transported to the laboratory in hermetically closed boxes at room temperature. Serum sampling procedures were approved by INTA’s National Committee for the Care and Use of Experimental Animals (CICUAE) through protocol No.25/2013, following biosafety criteria and animal welfare standards dictated by national and international regulations and reviewed by CICUAE.

### 2.3. Cells and virus

FMDV A24/Cruzeiro’s capsids that are more stable than those of the O1/Campos strain. Since the use of antigen with different stabilities can affect both the vaccine-induced immune response and the reliability of the assay used to measure those antibodies (16), FMDV-specific responses were assessed against both viral strains. Inactivated and concentrated preparations of both FMDV strains were prepared as described before (17, 23). Baby hamster kidney (BHK) cells, strain 21, clone 13 (ATCC, INTA’s repository) were maintained as described previously and used to grow the vaccine virus strains provided by SENASA (23, 24).

### 2.4. Animals and experimental design

Serum samples were obtained in 2013 from water buffaloes (*Bubalus bubalis*) located in two different farms (A, *n* = 43; and B, *n* = 48) in the Province of Corrientes, Argentina. Animals were immunized as part of the national FMD vaccination program using a conventional tetravalent oil-adjuvanted commercial vaccine approved by SENASA. Adult animals had already been immunized from previous vaccination campaigns (A, *n* = 25; B, *n* = 33) while calves, with or without maternal antibodies, received a single immunization (A, *n* = 18; B, *n* = 15). Individual serum samples were taken at 0, 7, 14, 21, 30, 60, 90, and 120-days post-vaccination (dpv), aliquoted in 500 μL cryotubes and stored at −20°C until processed. Samples taken at 0 dpv were used to set-up the assays (15). All assays were performed following biosafety and blood samples were obtained following animal welfare regulations, according to protocol 25/2013 approved by the Institutional Committee for Use and Care of Experimental Animals (CICUAE) from the CICVyA-INTA.

### 2.5. Liquid-phase blocking ELISA

Total anti-FMDV O1/Campos and A/24 Cruzeiro antibody responses were assessed by Liquid-phase blocking ELISA (LPBE) performed as stated by the WOAH Manual (1), using strain-specific rabbit antisera to capture inactivated FMDV O1/Campos or A/24 Cruzeiro particles and antigen-specific guinea-pig antisera as detector antibodies, followed by an anti-guinea pig-HRP conjugated (KPL, MD, United States). Antibody titers were expressed as the reciprocal log_10_ of serum dilutions giving 50% of the absorbance recorded in the virus control wells without serum. Positive and negative controls used for LPBE have already been described before (25). This protocol is used for vaccine control and approval, and it is routinely performed at INTA.

### 2.6. Neutralization assay

FMDV-neutralizing antibodies in serum of vaccinated buffaloes were titrated by a conventional virus microneutralization test (VNT) using infective culture adapted FMDV O1/Campos and A/24 Cruzeiro strains (10^7^ TCID_50_/mL) on BHK-21 (23, 26). Sera from multi-vaccinated cattle were used as positive controls and sera from naïve cattle from FMD-free without vaccination areas. Control samples were validated by the National Sanitary Authority (SENASA) and extensively used at INTA as described (23, 26). Serum samples were defrosted and heated at 56°C for 30 min to inactivate complement before performing the assay. Neutralizing antibody titers were expressed as the log_10_ serum dilution neutralizing 50% of the virus inoculum, according to the method of Reed and Müench (27).

### 2.7. Single dilution indirect and avidity ELISAs

Assessment of FMDV-specific total IgG and IgG avidity using single dilution indirect ELISA was performed as described by Lavoria et al. (17) and adapted to buffalo samples according to Sala et al. (8), using serum samples stored at −20°C. Briefly, samples were run by duplicates diluted 1:50 (final dilution): one of the wells was washed with PBS and the other with PBS 6 M Urea to detach low-avidity binders. OD values for samples and controls were corrected by subtracting mean blank OD values (cOD), which were used without further calculations. Estimation of cut-off value corresponding to the EPP_75_ for VNT was established before, being OD = 0.5 for IE and OD = 0.4 for AE (15).

### 2.8. Data analysis

Antibody titers induced after vaccination and measured by LPBE and VNT were also referred to the EPP_75_ already established for the A24/Cruzeiro and O1/Campos strains. As it was mentioned before, the EPP estimates the likelihood that cattle would be protected after the homologous FMDV challenge based on the specific antibody titers measured before the challenge. EPP values for the A24/Cruzeiro and O1/Campos strains arise from correlations between LPB-ELISA (20, 21) or VNT (27, 28) titers obtained in vaccinated cattle at 60 dpv, and the *in vivo* challenge results obtained at 90 dpv by the PPG test, involving 16 vaccinated animals infected with the homologous strain. For both total and neutralizing FMDV-specific antibodies, EPP_75_ values serve as a reference of antibody titers at the population level associated to the protection against the homologous challenge with the A24/Cruzeiro or O1/Campos strains. These titers are 2.10 and 1.90 for LPBE, and 1.66 and 1.40 for VNT for FMDV A24/Cruzeiro and O1/Campos, respectively.

Kinetics of humoral responses against the O1/Campos and A24/Cruzeiro vaccine strains were assessed in serum samples at 0, 7, 14, 21, 30-, 60-, 90-, and 120-days post-vaccination (dpv). Results were analyzed by multivariate two-way ANOVA, followed by Bonferroni correction to compare between different time points. Sensitivity and specificity were estimated using ROC curves for the best-fit values for each individual assay as described before (18). Tables were built to identify the frequency of true positive and negative results, as well as false positive and negative results according to the VNT titers used as reference assay (“gold standard”).

Sensitivity was estimated as the probability that a test will indicate protected [true positive/(true positive + false negative)] while specificity depicts the fraction of those samples without protective VNT titers that will have a negative test result [true negative/(true negative + false positive)]. VNT results were used as the gold standard for all these analyses. Also, positive predictive values (PPV) were calculated as: [true positive/(true positive + false positive)], and negative predictive values (NPV) were calculated as: [true negative/(true negative + false negative)]. Concordance between VNT and the ELISAs was assessed using Kappa value, considering the protection cut-off value for each assay and strain (*n* = 728 samples). Confidence interval was 95% for all assessments. Graph-pad Prism 5.0, Med-Calc and Sigma-Stat software were used for these analyses.

## 3. Results

### 3.1. Kinetics of antibody response after vaccination

**Figure 1:**
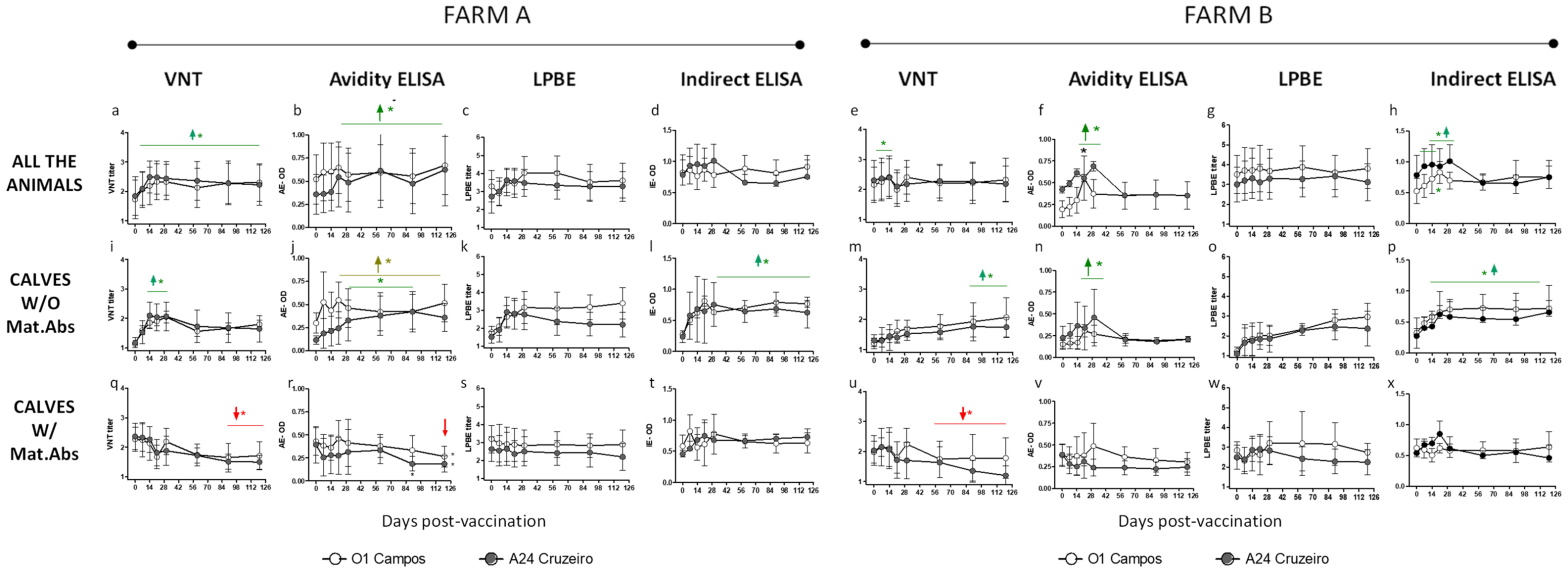
Kinetics of antibodies against A24/Cruzeiro and O1/Campos induced after a single vaccination in buffaloes. A single dose of a commercial FMD vaccine was applied in two different buffalo herds (Farms A and B) including adult animals and calves with or without maternal antibodies (CALVES W/Mat.Abs or CALVES W/O Mat.Abs respectively, as indicated Data from VNT, Avidity ELISA, LPBE and Indirect ELISA are depicted). Graphs have also been identified with letters (“**A–X**”) to facilitate identifying the graphs when reading the results section. For both strains, green horizontal lines and arrows indicate time-points with mean antibody titers significantly above those at 0 dpv. Red lines and arrows indicate time-points with mean antibody titers significantly below those at 0 dpv, also for both strains. Significant differences indicated with light brown lines and arrows are referred only to the O1 Campos strain. *p < 0.05.

The kinetics of the FMDV-specific antibody response against A24/Cruzeiro and O1/Campos strains induced after FMD vaccination were studied in two different farms using LPBE, VNT, IE, and AE (Figure 1). Previously, serum samples from both farms were assayed for antibodies against FMDV NSP, resulting negative in all cases (data not shown).

In farm A, the kinetics of specific antibodies was similar for both FMDV strains in the different assays, when considering all the animals as a single group. Serum antibody titers increased from 0 to 14 dpv and maintained similar levels from thereon (between 2 and 2.8 for VNT; above 3 for LPBE; over 0.6 for IE and AE). Although all the assays presented this tendency, only the VNT and AE values were significantly higher from 14 to 120 dpv when compared to the pre-immune values (Figures 1A,B).

Results were similar in Farm B. Neutralizing antibodies significantly increased their titers at 7 and 14 dpv compared to basal levels (0 dpv) for both strains (Figure 1E). Both the AE and IE assays also registered a significant increase in their titers from 14 to 30 dpv (Figures 1F,H). As observed in farm A, LPBE titers were high for both viruses and did not vary significantly from day 0 to 120 in Farm B (Figure 1G).

Both farms included adult individuals with detectable FMDV-specific antibodies at 0 dpv because of the previous FMD vaccinations. Only young animals (below 9 months of age) were primary vaccinated. Among those calves, there were animals with or without maternal antibodies at the time of vaccination. To get an accurate insight of the antibody response in the presence or absence of maternal antibodies we analyzed antibody levels separately in these two groups.

Vaccinated calves without maternal antibodies from farm A elicited a rapid neutralizing antibody response with peak titers from 14 to 30 dpv against both the A24/Cruzeiro and O/Campos strains. VNT titers slightly decayed thereafter, though mean values were above the EPP_75_ cut-off for both viral strains during the tested period (Figure 1I). Avidity maturation measured by the AE showed a gradual increase over time. Avidity levels were higher to basal levels (0 dpv) starting at 21 dpv for the O1/Campos strain and from 30 dpv for the A24/Cruzeiro strain (Figure 1J). LPBE titers presented a similar trend with higher antibody levels against O1/Campos than A24/Cruzeiro strain, though differences were not statistically significant at any time-point (Figure 1K). The Indirect ELISA depicted a traditional dose–response antibody curve for both strains, resulting significantly above pre-immune levels from 21 dpv, with peak titers at 30 dpv that remained high thereafter (Figure 1L).

In farm B, calves without maternal antibodies developed a delayed kinetics response when assessed by VNT and LPBE (Figures 1M,O), while the same samples assessed by IE exhibited a typical dose–response curve (Figure 1P). VNT titers were significantly above the initial values only at 90 and 120 dpv (*p* < 0.05; Figure 1M). Avidity matured around 30 dpv but only low avidity antibodies were found thereafter for both strains (Figure 1N).

In both farms, calves vaccinated in the presence of maternal antibodies displayed a decline in the neutralizing antibody response after vaccination against the O1/Campos and A24/Cruzeiro strains (Figures 1Q,U). This was also observed by the AE, but only for the A24/Cruzeiro strain. The decrease in the antibody titers detected by both assays were significantly different from basal levels (0 dpv) only at 90 and 120 dpv (*p* < 0.05; Figures 1R,V). This decay was not observed for any of the FMDV strains in any of the farms when titrated using the LPBE (Figures 1S,W). Interestingly, only the AE detected a reduction in the avidity of antibodies against A24/Cruzeiro in both farms, while in farm B only VNT detected a decay in A24/Cruzeiro antibodies.

**Figure 2:**
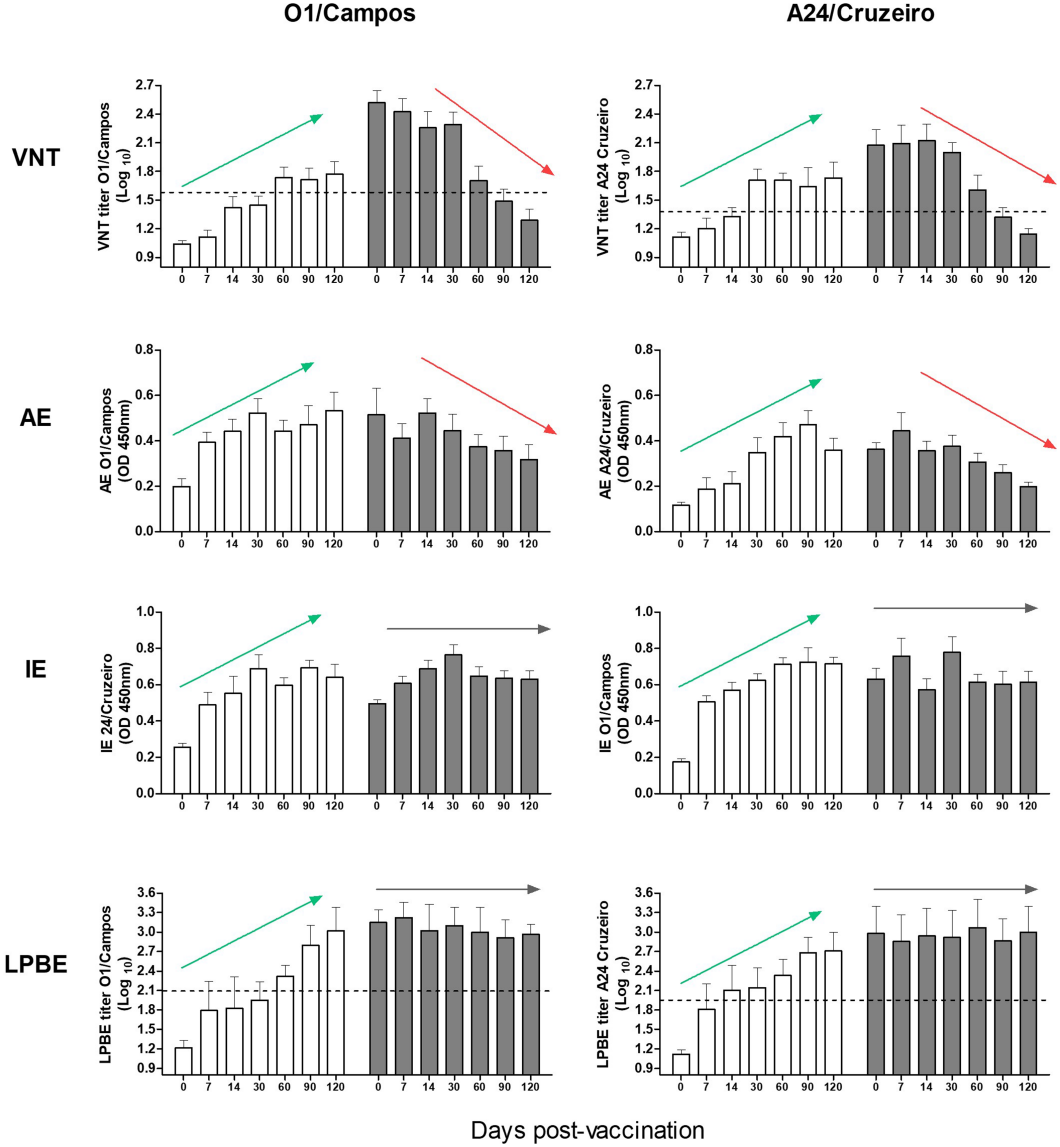
Immune response to vaccination in calves with or without maternal antibodies. Antibody titers from both farms were simultaneously analyzed by VNT, AE, IE, and LPBE for the A24/Cruzeiro and O1/Campos strains, as indicated. Bars represent mean values +/− SD in calves with (gray) or without (white) maternal antibodies at 0 dpv. Dotted lines indicate the corresponding EPP_75_ cut-off values estimated in cattle for the VNT and LPBE assays. Arrows highlight the overall tendency of the antibody kinetics (green for increasing trends, red for decreasing trends and gray for unvarying tendencies).

The results of serology using the different assays were then analyzed altogether, considering both farms, segregated by virus strain and by the presence or absence of maternal antibodies at 0 dpv (Figure 2). These charts clearly show that VNT, and AE provide similar information, while LPBE and IE cannot identify the antibody decay due to the presence of maternal antibodies. These would indicate that these tests may not be appropriate for assessing vaccine-induced antibody responses in young animals in areas where compulsory vaccination is applied, and maternal antibodies may be present at the time of vaccination. At 90 dpv, neutralizing antibody titers in calves with maternal immunity (dark gray bars; Figure 2) were below the EPP_75_ cut-off value, meaning that these calves might not be fully protected 3 months after vaccination.

Considering all the assays, except IE, it was evident that antibody levels against FMDV O1/Campos were higher than those for the A24/Cruzeiro strain.

### 3.2. Concordance between VNT and ELISAs

**Figure 3:**
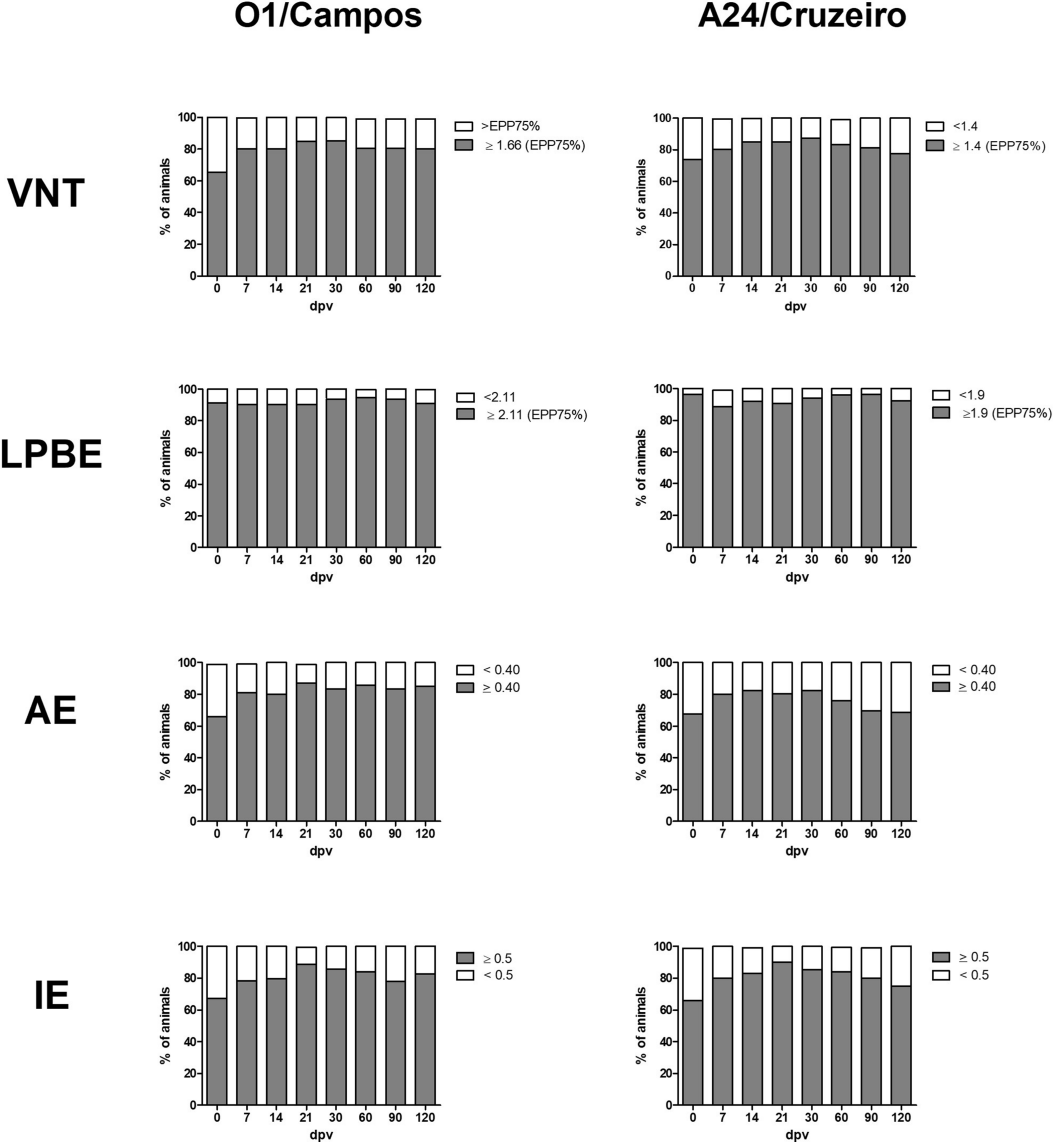
Antibody levels at the different days post-vaccination (dpv) according to their cut-off values for A24/Cruzeiro or O1/Campos Serum samples from both farms were grouped for each serological test according to the corresponding EPP_75_ against the A24/Cruzeiro or O1 Campos strains. Gray bars indicate the frequency of samples above the cut-off value, and white bars indicate frequency of samples below the EPP_75_ for each assay.

A total of 728 samples, 384 from farm A and 344 from farm B, were analyzed using the different ELISAs and by VNT against the O1/Campos and A24/Cruzeiro strains. For each strain, the neutralizing antibody titers corresponding to the EPP_75_ were used as cut–off values in these assays. Considering VNT as the reference “gold standard” assay, with a cut-off of 1.66 for O1/Campos and 1.4 for A24/Cruzeiro strains, we analyzed the concordance of each assay by computing the Kappa value (Table 1). Figure 3 shows the percentage of animals that would be categorized as protected (gray) or not-protected (white) considering the corresponding EPP_75_ as the cut-off values. VNT and AI results presented similar progression for both strains. Similarly, total antibodies measured by LPBE paralleled those measured with the indirect ELISA.

**Table 1:** Performance of the avidity ELISA (AE), Indirect IgG ELISA (IE) and Liquid-Phase Blocking ELISA (LPBE) compared to the virus neutralization test (VNT).

		Vaccine strain		
Assay	Analysis	O1/Campos	A24/Cruzeiro	Concordance with the VNT
AE	Sensitivity	0.87	0.86	
	Specificity	0.93	0.94	
	Accuracy	0.88	0.92	
	Kappa	0.78	0.76	Considerable
IE	Sensitivity	0.90	0.92	
	Specificity	0.81	0.90	
	Accuracy	0.88	0.92	
	Kappa	0.68	0.75	Considerable
LPBE	Sensitivity	0.88	0.90	
	Specificity	0.67	0.66	
	Accuracy	0.83	0.85	
	Kappa	0.55	0.56	Moderate

Sensitivity, specificity accuracy and concordance (Kappa value) were calculated for each serological assay (results from all the samples, *n* = 728), indicating the level of concordance according to this coefficient.

**Figure 4:**
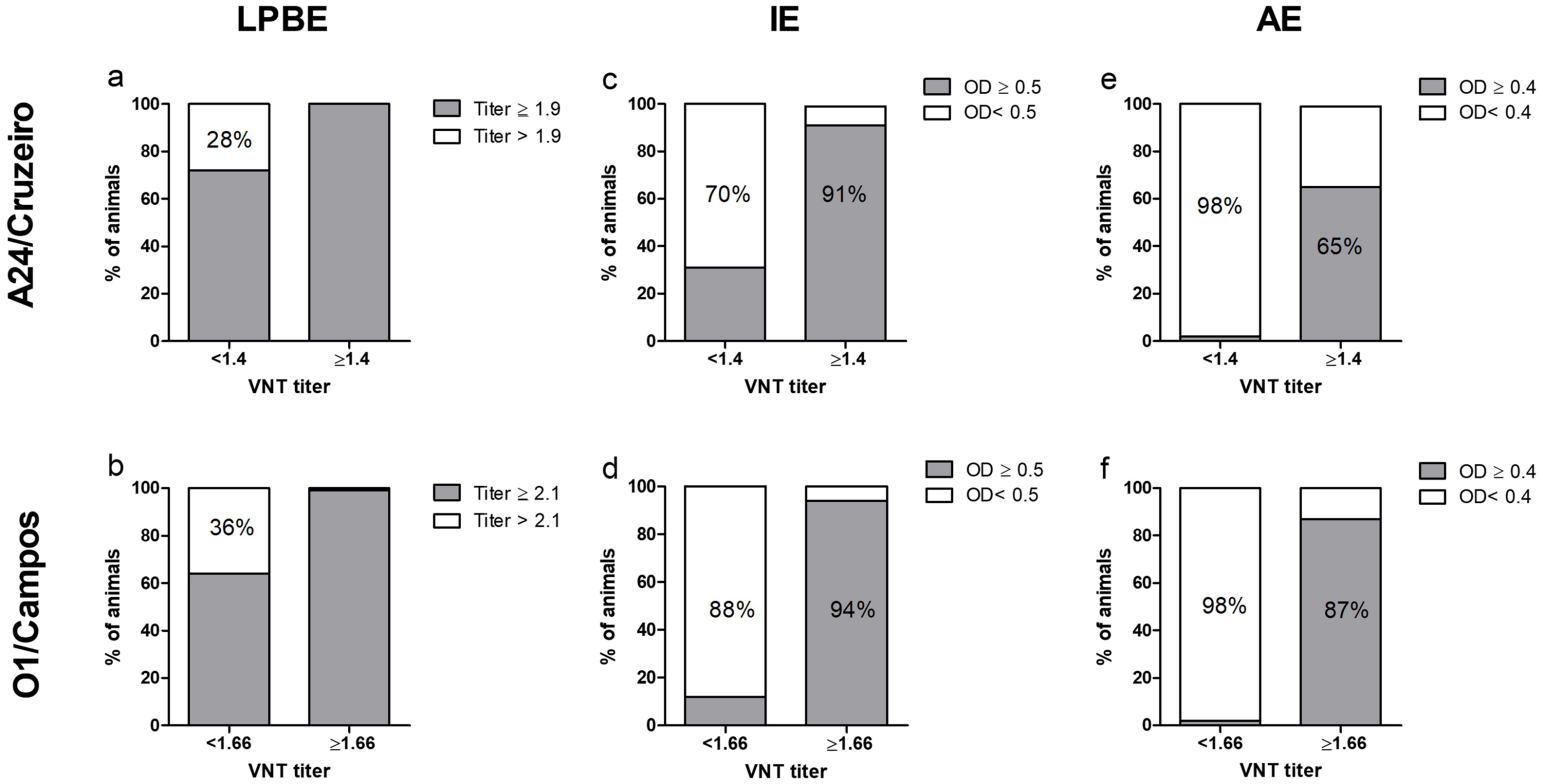
Sample categorization by the different serological assays according to the VNT EPP_75_ values. Samples from both farms were grouped according to the EPP_75_ VNT titers for the A24/Cruzeiro (log_10_ 1.4, panels **A,C,E**) and O1/Campos (log_10_ 1.66, panels **B,D,F**) strains. Samples were classified within each VNT-titer subgroup according to the corresponding cut-off values for each of the serological assays, as indicated in each panel. Values within the bars indicate the percentage of samples correctly classified by each test using the EPP_75_ VNT titers as a reference.

ELISA results were then sorted according to each sample’s VNT titer, and the percentage of samples correctly classified by each assay was calculated (Figure 4). LPBE overestimated protective antibodies titers, since only ~30% of the negative VNT samples were correctly classified using this method (Figures 4A,B). On the contrary, over 90% of negative VNT samples were correctly identified by the AE (Figures 4E,F), while the IE test yielded intermediate values (70% and 88%, for O1/Campos and A24/Cruzeiro, respectively). Regarding the high-titer VNT samples, the IE assay was the most accurate test with over 90% of correctly classified samples for both strains (Figures 4C,D).

Sensitivity, specificity, overall accuracy, and concordance with VNT were also calculated for each ELISA test for both virus strains (Table 1). We found a considerable concordance between the AE and VNT (Kappa value = 0.76 and 0.78 for A24/Cruzeiro and O1/Campos strains, respectively), while there was only a moderate concordance with LPBE (0.56 and 0.55 for A24/Cruzeiro and O1/Campos, respectively). Interestingly, considering only OD values obtained in the indirect ELISA, concordance was similar to that of AE and VNT (Kappa value = 0.75) for A24/Cruzeiro but lower (0.67) for O1/Campos strain. Both IE and AE achieved similar diagnostic accuracy values: while both presented similar sensitivity, the AE assay was more specific than the IE.

The AE was the only assay that could predict with high certainty protective levels of antibodies, due to the higher specificity of the test. In contrast, the other ELISA tests may overestimate antibodies, being LPBE the worst predictor. Due to the high specificity of the AE, the probability of a negative result to be truthful is over 90%. Using IE allows better detection of VNT positive samples (over 90% for both strains) than the other serological assays.

These results show that measurement of the FMDV-specific avidity of the vaccine-induced antibodies can be a good replacement of the VNT, as it is shown by its high concordance with this highly specific biological assay. The assessment of protective antibodies may be complemented by using the IE to increase the overall sensitivity in the detection of vaccine induced anti-FMDV antibodies in buffalo’s serum samples.

## 4. Discussion

The role of water buffaloes in FMDV epidemiology as one of the major hosts of FMDV that can develop persistent asymptomatic infection (8, 29, 30) highlights the importance of sustaining surveillance on the antibody response elicited by vaccination in these animals.

Argentinean water buffalo heads are located along the borders with Paraguay and Brazil, in areas close to those that will stop vaccinating. Venezuela has the largest population of water buffaloes in the region and is the only country with unrecognized WOAH status. Meanwhile, South America is currently moving toward FMDV eradication which requires the application of suitable tools to control the immunological status of buffalo populations. This same scenario can be found in other parts of the world where buffaloes are key actors of FMDV epidemiology (8, 30).

Based on this background, several papers studied the epidemiological role of buffalo’s populations in the emergence and persistence of FMDV in the field (31), as well as the pathogenesis of the FMDV infection in these species, focusing on the transmission (32), disease symptoms (33, 34), persistence (30, 35, 36) and viral dynamics (37). However, few reports informed about the FMDV-specific antibody responses in buffaloes, basically studying the neutralizing antibody responses by VNT in naïve and vaccinated animals after FMDV infection (32, 38).

In this regard, there is gap in the knowledge on how serological assays that measure antibodies against capsid proteins perform with buffalo samples and which would be the most reliable test to substitute VNT for field surveillance. This study is the first one reporting the kinetics of the antibody response elicited by vaccination in *Bubalus bubalis*, considering the presence of animals with maternal antibodies in the herd, a regular scenario in areas where vaccination is applied. We confirmed there is a high concordance between the avidity ELISA and VNT results, moreover, avidity of antibodies measured by ELISA and the VNT titers produced similar antibody decay curves when calves were vaccinated in the presence of maternal antibodies, which underpin the use of Avidity ELISA (AE) as an alternative to the VNT.

VNT results inform on the biological activity of antibodies which has been related to protection provided by FMD vaccines (24), however, this assay entails several drawbacks that have already been summarized (39). ELISAs are always preferred over VNT for serology monitoring. The first ELISA measuring antibodies against capsid antigens used in our region was the LPBE, that quantifies total antibody levels. This test relies on the development and standardization of capture and detector antibodies that need to be produced for each individual virus strain. Furthermore, the inactivated virus suspension used for this assay possesses an unknown content of whole and disassembled particles that can impact in the assay’s performance (15). Cross-serotype reactive antibodies have been described when using this method (40). We proposed before (41) that the biological features of the humoral responses in buffaloes may also interfere with LPBE results, causing an inaccurate assessment of the vaccine-induced antibody responses, especially due to false positive results. The data presented here confirm the insufficient performance of LPBE with buffalo samples, at least when using the cut-off values validated for cattle. Considering all the data and splitting the results based on the VNT EPP_75_ threshold, we verified that when the serum neutralizing antibody titers are low, the LPBE yielded high values and barely manages to classify correctly around 30% of the samples. If LPBE is used with buffalo sera, a new cut-off should be estimated as the cut-off set up for cattle does not minimize the number of false positive results, and it is not accurate enough for replacing the VNT.

To overcome the technical and practical drawbacks of using LPBE for buffalo’s samples, we developed indirect ELISAs that use purified 140S particles as capture antigen (41). In that first study we analyzed the accuracy of the AE, IE and LPBE for testing buffalo samples and proposed that the use of avidity ELISA can be more reliable to assess protective antibodies than other techniques that only measure total antibody levels. A recent study concurred on the potential efficiency of avidity ELISA for the assessment of vaccine-induced antibodies in buffaloes. In this study we confirmed these observations (19).

Using indirect whole-virus based ELISAs (AE and IE) have several technical advantages we have analyzed before (18). From a practical point of view and in our hands, a single antigen batch consisting in 30 mL of a PEG-concentrated inactivated virus batch yielded about 3 mg of purified 140S particles, enough for preparing 300 plates (10 μg per plate) and testing 120,000 serum samples. A single trained operator can run up to 10 plates (400 samples) in less than half-day of work, producing results that are objective and traceable, suitable for high-throughput field surveillance. In this study we demonstrated that IE is more sensitive than AE, being AE highly specific. The combined used of IE and AE, that can be performed by running duplicate wells in the same plate can produce the results that are concordant with the VNT, as described before (15).

Another important information provided by this study is that, as observed in cattle, neutralizing antibodies are not elicited after primary vaccination in calves with maternally derived antibodies. Assays measuring total antibodies (IE, LPBE) cannot identify this lack of novel vaccine-induced humoral immune response. Interestingly, avidity of antibodies and VNT produced similar antibody decay curves when calves were vaccinated in the presence of maternal antibodies, which, together with the high concordance between this assay and the VNT results, supports the use of AE as an alternative to the VNT. It is worth noting that 3 months after primary vaccination, calves` antibody levels were below the EPP_75_ cut-off value for VNT titers for both viruses and might therefore be susceptible to FMDV infection. Based on these data, re-vaccination of young buffaloes when maternal antibodies have weaned should be considered.

This is the first time a kinetics of total IgG, total antibodies, neutralizing antibodies, and avidity maturation are studied in buffalo herds in field conditions, considering pre and re-vaccination, and primary vaccination in the presence or absence of maternal antibodies. Similarly, neutralizing antibody responses increases after infection in African buffaloes by 14 dpv (VNT titer ~2) and maintained thereafter (8).

It is important to note that specific humoral immunity is induced rapidly after vaccination and that antibody levels are maintained over-time. On the contrary, neutralizing antibody responses decline after vaccination in the presence of maternal immunity, and a similar trend was also verified in the avidity maturation capacity of the vaccine-induced antibodies, as previously described for cattle by our group (42).

The implementation of a systematic vaccine serosurveillance policy is important to successfully eradicating FMDV. The selection of the most appropriate assay to be used for this purpose should consider not only the concordance with VNT but also the test’s sensitivity and specificity for each species, which may require producing fit-for-purpose cut-off values. In a previous study using a lower number of samples we needed to increase the number of tests to improve concordance with the VNT, used as the gold standard reference assay (15). In this study with a larger number of data being analyzed, concordance with the VNT improved from moderate to considerable by using a sole single dilution assay. It is important to note that the ease and high throughput of the selected assays are also paramount for a successful implementation of massive surveys. The value of sensitivity and specificity is not the final measure of test accuracy. Predictive values must also be considered when developing any testing strategy. Understanding the meaning of results and limitations of each test is critical to using testing as a tool to make policy or operational decisions.

We conclude that avidity of anti-FMDV antibodies that can be measured using a simple single-dilution ELISA provides similar results as the VNT and might replace this reference assay for vaccine-immunity monitoring in vaccinated buffalo herds, irrespectively of the presence or absence of maternally derived antibodies. These results are important to assist regional efforts for working on risk scenarios and organizing follow-up tools to enable a big part of South America to acquire the WAHO FMD-free without vaccination status.

## Data availability statement

The raw data supporting the conclusions of this article will be made available by the authors, without undue reservation.

## Ethics statement

The animal study was approved by the National Committee for Care and Use of Experimental Animals (CICUAE), Protocol No.25/2013 CICUAE - INTA. The study was conducted in accordance with the local legislation and institutional requirements.

## Author contributions

AC conceived and planned the experiments and took the lead in analyzing the results, preparing the graphs, and writing the manuscript together with DP-F. DP-F and AC received the funding for the experimental work. JS designed and carried out most of the experiments, including vaccination, sampling, and laboratory testing as part of his PhD thesis, directed by AC and supervised by SC. FM and MM assisted in the set-up and performance of the ELISAs, including virus purification and QC. All authors contributed to the article and approved the submitted version.
